# Immune Signatures in Post-Acute Sequelae of COVID-19 (PASC) and Myalgia/Chronic Fatigue Syndrome (ME/CFS): Insights from the Fecal Microbiome and Serum Cytokine Profiles

**DOI:** 10.3390/biom15070928

**Published:** 2025-06-25

**Authors:** Martin Tobi, Diptaraj Chaudhari, Elizabeth P. Ryan, Noreen F. Rossi, Orena Koka, Bridget Baxter, Madison Tipton, Taru S. Dutt, Yosef Tobi, Benita McVicker, Mariana Angoa-Perez

**Affiliations:** 1 R&D Department, John D. Dingell VAMC, 4646 John R Street, Detroit, MI 48201, USA; nrossi@wayne.edu (N.F.R.); maperez@med.wayne.edu (M.A.-P.); 2 Department of Physiology, Wayne State University School of Medicine, Detroit, MI 48301, USA; hu8715@wayne.edu (D.C.); orena.koka@med.wayne.edu (O.K.); 3 Department of Environmental and Radiological Health Sciences, School of Health System, Public Health, College of Veterinary Medicine and Biomedical Sciences, Colorado State University, Fort Collins, CO 80523, USA; e.p.ryan@colostate.edu (E.P.R.); bridget.baxter@uchealth.org (B.B.); maddy_tipton@yahoo.com (M.T.); taru.dutt@colostate.edu (T.S.D.); 4 Department of Internal Medicine, University of Nebraska Medical Center, Omaha, NE 68005, USA; bmcvicker@unmc.edu

**Keywords:** COVID-19, Long COVID, PASC (Post Acute Sequelae of SARS-CoV-2 infection) or post-acute sequelae of COVID-19, stool microbiome, cytokines, p87, Adnab-9, chronic fatigue, innate immune system, database, post-viral symptoms, chronic fatigue syndrome, myalgic/encephalitis, Epstein Barr Virus, cytomegalovirus, oligo-A-synthetase, animal models

## Abstract

While there are many postulates for the etiology of post-viral chronic fatigue and other symptomatology, little is known. We draw on our past experience of these syndromes to devise means which can expose the primary players of this malady in terms of a panoply participating biomolecules and the state of the stool microbiome. Using databases established from a large dataset of patients at risk of colorectal cancer who were followed longitudinally over 3 decades, and a smaller database dedicated to building a Long PASC cohort (Post-Acute Sequelae of COVID-19), we were able to ascertain factors that predisposed patients to (and resulted in) significant changes in various biomarkers, i.e., the stool microbiome and serum cytokine levels, which we verified by collecting stool and serum samples. There were significant changes in the stool microbiome with an inversion from the usual *Bacillota* and *Bacteroidota* species. Serum cytokines showed significant differences in MIP-1β versus TARC (CC chemokine ligand 17) in patients with either PASC or COVID-19 (*p* < 0.02); IL10 versus IL-12p70a (*p* < 0.02); IL-1b versus IL-6 (*p* < 0.01); MCP1 versus TARC (*p* < 0.03); IL-8 versus TARC (*p* < 0.002); and Eotaxin3 versus TARC (*p* < 0.004) in PASC. Some changes were seen solely in COVID-19, including MDC versus MIP-1α (*p* < 0.01); TNF-α versus IL-1-β (*p* < 0.06); MCP4 versus TARC (*p* < 0.0001). We also show correlates with chronic fatigue where an etiology was not identified. These findings in patients with positive criteria for PASC show profound changes in the microbiome and serum cytokine expression. Patients with chronic fatigue without clear viral etiologies also have common associations, including a history of tonsillectomy, which evokes a likely immune etiology.

## 1. Introduction

It would be unlikely that a single laboratory would be able to perform multiple assays commensurate with the knowledge and skill required to inform a wide array of germane testing leading to successful integration of these variable branches of endeavor. In this paper, multiple disciplines have seamlessly cooperated to provide a thorough understanding of the underpinnings of the variable conditions of PASC (Post-Acute Sequelae of COVID-19). Despite the untimely passing of our champion of the microbiome, Dr. D. Kuhn (OBM), his colleagues at WSU have continued his legacy. Below, we describe he methodology and results of an exhaustive effort linking PASC to the microbiome. This could not have come about without the vision and foresight of our colleagues at the University of Colorado, who established a biorepository of material that encompassed a longitudinal collection of samples of blood and stool from PASC and COVID-19 patients that was indispensable to this effort and without which this manuscript would not have been realized. It would be unthinkable to try to bring to the reading public a work that did not involve the role of cytokines that likely underlie the pathways that subtend the final common pathway of reaction in the face of infection of both COVID-19 [1] and other viral infections. This was expeditiously performed by Dr. McVicker of the Department of Internal Medicine, University of Nebraska Medical Center, Omaha, and was part of the triumvirate that included contributions by Dr. Rossi of the Department of Physiology at WSU. This effort began in the wave of chronic atypical illness that we described in 1982 [2] and the role of interferon immunity that we were able to implicate using an assay to show that the activity of 2′-5′ oligo-A-synthetase (OAS1) with IgM activity was associated with Epstein-Barr Virus (EBV) infection [3]. At the time, there was an acceleration of articles with a larger number of patients [4], which ultimately led to an internationally attended group of researchers at the National Institutes of Health (NIH) [5]. Due to the stress imposed by the growing number of affected individuals, a trial of acyclovir was inaugurated [6] without much success. Moving ahead to the COVID-19 pandemic, workers found an ancient isoform of OAS1 that was protective in certain individuals [7]. Just before this, a comprehensive review of myalgic encephalomyelitis/chronic fatigue syndrome was published, bringing these disease isoforms up to date [8,9,10,11,12,13,14,15,16,17,18]. In the last 4 years, PASC was related to EBV reactivation [19], and risk factors were explored [20,21,22]. We and others also established methods of cognitive deficit evaluation that were applicable to PASC [23,24]. Cardiac and virologic issues had been studied in 2003 [25] that extended to Veterans health, and later, to PASC [26,27]. The delineation of the length of Long COVID was also debated [28], accompanied by the linkage to microbiome dysbiosis [29] and epitheliopathy [30]. Antibody response was associated with outcomes [31], and PASC publications were common [32,33,34,35]. Studies from the animal virologic literature suggested non-human primate coronaviruses may have had a parallel to those in humans [36], and the urgent search for new anti-COVID-19 medications, biomarkers, and vaccines was being conducted at a rapid pace [37,38,39,40,41,42,43].

## 2. Materials and Methods

### 2.1. Human Stool, Blood Plasma Sample Collection and Storage

Stool samples and plasma blood were collected from participants enrolled in the Northern Colorado COVID-19 Biobank (NCT04603677) using sterile, pre-labeled polypropylene containers supplied by the research team. Participants were given detailed written and verbal instructions to ensure proper collection, including avoiding contamination with urine or water (https://pubmed.ncbi.nlm.nih.gov/34256735/; https://pubmed.ncbi.nlm.nih.gov/34769566/; https://pubmed.ncbi.nlm.nih.gov/34769566//; https://pubmed.ncbi.nlm.nih.gov/38327763/, accessed on 15 May 2025). Immediately after collection, samples were placed in insulated coolers with ice packs and transported to the laboratory within 2 h to minimize degradation of microbial and metabolite profiles. Upon arrival at the laboratory, all samples were processed in a biosafety cabinet under aseptic conditions. Each specimen was thoroughly homogenized to ensure consistency, and stool aliquots (200–300 mg per vial) were prepared in sterile, DNA/RNA-free cryovials. These aliquots were immediately transferred to long-term storage at −80 °C. To preserve sample integrity, repeated freeze–thaw cycles were avoided by storing multiple aliquots per sample. All samples were logged with unique identifiers to ensure traceability throughout the study. Sample processing and storage followed standard operating procedures (SOPs), which were developed to maintain consistency across all time points and participants. All stool samples were lyophilized and aliquoted into 100 mg vials, then shipped to Michigan for further analysis.

#### 2.1.1. Gut Microbiome Profiling

DNA from fecal samples was extracted for controls and Long COVID samples using QIAamp PowerFecal DNA kits, and sample DNA concentrations were determined using a Qubit 4 Fluorometer, as previously described [44]. Samples were sequenced on an Illumina MiSeq system with Illumina reagents using a 2 × 250 cycle V2 kit following sequencing procedures previously detailed [45]. The primers targeted the V4 region of the 16S rRNA gene (forward primer: 5′-GTGCCAGCMGCCGCGGTAA-3′; reverse primer: 5′-GGACTACHVGGGTWTCTAAT-3′).

#### 2.1.2. Bioinformatics and Statistical Analysis

For this analysis, 16S rRNA gene sequences were clustered into amplicon sequence variants (ASVs) using the Divisive Amplicon Denoising Algorithm (DADA2) pipeline [46] to obtain samples that were merged, denoised, chimera-free, as previously reported by our group [47,48], with the exception that forward reads were truncated at 230 bases and reverse reads at 55 bases. ASVs that were likely to be potential background DNA contaminants were identified with the R package decontam version 1.20.0 [49]. Davis using the “IsContaminant” method, which identifies contaminant sequence features and allows for their removal. An ASV was deemed a contaminant if it had the following characteristics: a decontam P score ≥ 0.5; presence in 75% of negative controls with an overall average relative abundance of at least 1.0%; and a greater than average relative abundance in controls compared to fecal samples. Based on these criteria, 12 ASVs were identified as DNA contaminants and were removed from the data set. These 12 ASVs had a total abundance of 0.026% in the dataset and corresponded to the following taxa: *Caldalkalibacillus* (3 ASV’s), *Bacillaceae* (3 ASV’s), *Halomonas* (3 ASV’s), *Ornithinibacillus* (1 ASV), *Nesterenkonia* (1 ASV), and *Xanthomonadales* (1 ASV).

Three datasets contained 1652 ASVs after the contaminant ASVs were removed. Analysis was performed on a subsampled count of 65,633 sequencing reads per sample, which was the lowest sequence depth to ensure an unbiased analysis. All samples had a Good’s coverage score of ≥99.8%. Microbial alpha-diversity was characterized using the Chao1, Shannon, and inverse Simpson indices, whereas beta-diversity was assessed using the Jaccard and Bray-Curtis similarity indices. Variation in the bacterial profiles of fecal samples from the different groups (controls, and Long COVID groups) were analyzed using three-way ANOVA in GraphPad Prism (version 10). β-diversity was statistically evaluated with permutational multivariate analysis of variance (PERMANOVA) and visualized with principal coordinates analyses (PCoA). Differential relative abundance of bacterial ASVs between controls and COVID-19 subjects were stratified based on sex and visit using a negative binomial model in the Microbiome Multivariable Associations with Linear Models 2 (MaAsLin2, R package v 1.14.1) [50,51]. A minimum prevalence of 0.25 was used, and multiple comparisons were adjusted using the Benjamini-Hochberg method. Values of *p* < 0.05 were deemed to be statistically significant.

#### 2.1.3. Metadata and Sample Collection

Stool samples were collected from 19 healthy controls (9 males, 10 females) and 13 Long COVID patients (6 males, 7 females) for microbiome analysis. Long COVID patients provided stool samples at two different visits (26 samples in toto), while controls contributed samples at a single time point (19 samples). COVID-19 samples for Visit 1 were collected between days 1 and 34 following the confirmed positive diagnosis for COVID-19. Samples for Visit 2 were collected during a time frame ranging from day 49 to 310 post-diagnosis. For each participant, demographic information, such as age, sex, symptomatology, and hospitalization status, were recorded. Additionally, for Long COVID patients, data were gathered regarding their clinical history, including the time of COVID-19 diagnosis, severity of symptoms, and treatments received. Patients were rated as: No PASC = 0; presence PASC = 1; fatigue, dyspnea, joint pain, chest pain, forgetfulness or absent minded, difficulty concentrating or confusion. To be included there must have been one symptom at one visit.

#### 2.1.4. Human Cytokine and Chemokine Analysis

Assays were performed as per the manufacturer’s protocols and analyzed on the MESO QuickPlex SQ 120 imager (Meso Scale Discovery, Rockville, MA, USA). Values were log-adjusted. This system has also been used by others investigating plasma and derived extracellular vesicles [50]. (PBL assay science,131 Ethel Road West Suite 6, Piscataway, NJ 08854, USA). Cytokine and chemokines were measured in serum or plasma samples using V-Plex Proinflammatory Panel 1 (K15049D) or Chemokine Panel 1 (K15047G) from Meso Scale Discovery.

Cytokine and chemokines were measured in serum or plasma samples using V-Plex Proinflammatory Panel 1 (K15049D) or Chemokine Panel 1 (K15047G) from Meso Scale Discovery (Rockville, MA, USA). Assays were performed as per the manufacturer’s protocols and analyzed on the MESO QuickPlex SQ 120 imager (Meso Scale Discovery). Values were log-adjusted. This system has also been used by others investigating plasma and derived extracellular vesicles. (PBL assay science, 131 Ethel Road West Suite 6, Piscataway, NJ 08854, USA) (https://www.mesoscale.com/~/media/files/scientific%20poster/multiplexed-electrochemiluminescent-immunoassays-intact-extracellular-vesicles-isev-2019.pdf, accessed on 13 May 2025).

Methodologic Amalgamation of a variety of Chronic Atypical Illness Bearing the Hallmarks of Chronic Fatigue.

Chronic forms of various diseases are in the medical literature, and many are of an inflammatory nature, persisting for many years. They can have prionic, viral, bacterial, or fungal etiology, but are not limited to these. Most are poorly understood. In modern times, the complex of myalgic encephalitis/chronic fatigue syndrome (CFS/ME) continues to be of interest. Despite a burgeoning body of literature, including a dedicated journal, CFS/ME remains inscrutable, with no universally accepted etiology and syndromic definitions that are still being researched. In this paper, we compare and contrast the ongoing clinical entity arising from acute COVID-19 infection variously known as Long COVID, long-haulers and such like monikers, with the described chronic Epstein Barr viral syndrome and CFS/ME.

#### 2.1.5. Permissions and Approvals

Colorado State University’s Research Integrity and Compliance Review Office Institutional Review Board (IRB; protocol ID 20-10063H, 12 November 2020) approved the biorepository, as well as the Colorado Health System IRB (Colorado Multiple IRB 20-6043, 2 June 2020) and is registered with ClinicalTrials.gov (NCT04603677). All enrolled participants provided written informed consent. The larger database was approved by the IRB (institutional review board) and Human Investigation Committee of Wayne State University. The approval numbers are #070700MP4F and #H 09-62-94, and the approval dates are 13 September 2006 and 17 August 2000, respectively. All patients gave informed consent.

#### 2.1.6. Statistical Analysis

Three-way ANOVA analyses were performed to examine the main effects of group (control and COVID-19, sex (males and females) and visit (Visit 1 or Visit 2) on microbial alpha-diversity. Alpha-diversity was measured with three indices, including Chao-1 Shannon and inverse Simpson. None of these measures showed statistically significant differences when comparing control and Long COVID, neither by sex nor visit.

## 3. Results of PASC Patients

**Figure 1:**
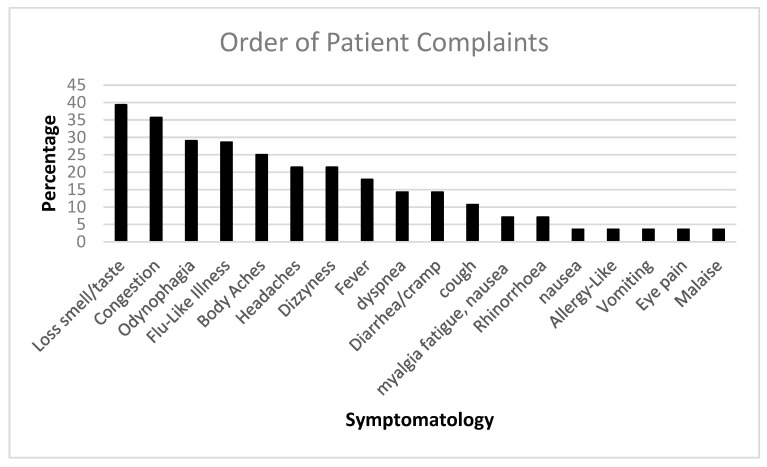
The bar diagram shows the initial COVID-19 complaints of patients who contracted PASC.

**Figure 2:**
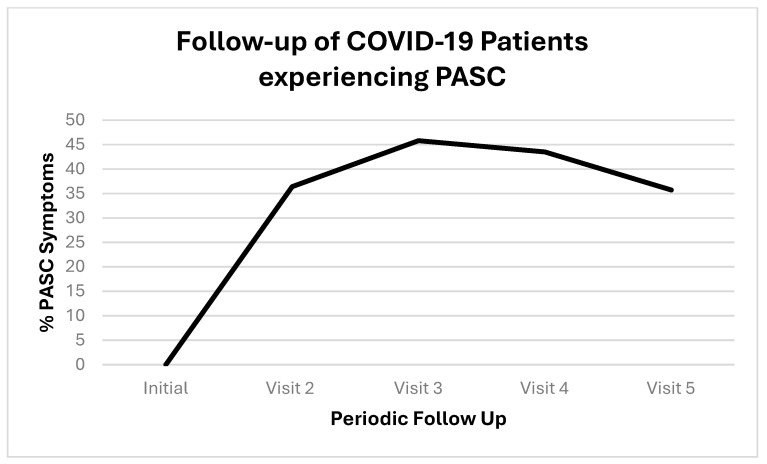
Timeline of patients who contracted PASC. Proportions are represented by positives (+) or negative (−); the positives are represented as percentage with both as the total denominators.

We followed up with PASC patients over time. Figure 1 shows the initial COVID-19 complaints of patients who contracted PASC. Figure 2 shows the progression of PASC symptomatology over time, with approximately 2.5 months between visits (Table 1 and Table 2).

### 3.1. Gut Microbiota Alpha- and Beta-Diversity

Three-way ANOVA analyses were performed to examine the main effects of group (control and COVID-19), sex (males and females), and visit (Visit 1 or Visit 2) on microbial alpha-diversity. Alpha-diversity was measured with three indices, including Chao-1. None of these measures showed statistically significant differences when comparing control and long COVID-19, neither by sex nor visit.

**Figure 3:**
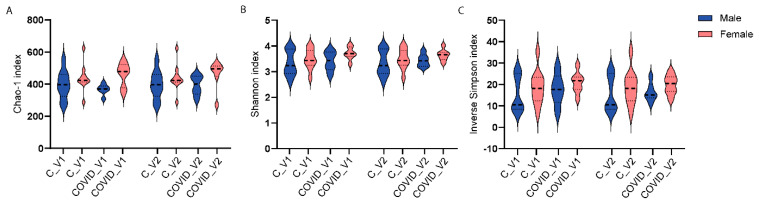
Alpha-diversity indices Chao1 (**A**), Shannon (**B**), and inverse Simpson (**C**) of 16S rRNA gene profiles of fecal samples from controls (C) and long COVID-19 (COVID) participants for Visit 1 (V1) and Visit 2 (V2).

**Figure 4:**
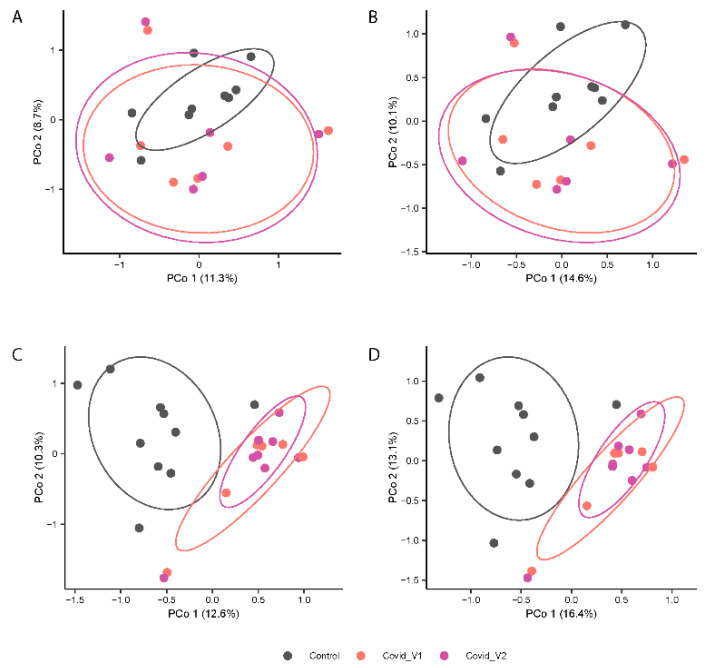
Principal Coordinates Analysis (PCoA) Depicting the Variation in Microbial beta-diversity using the Jaccard index (**A**,**C**) and Bray-Curtis index (**B**,**D**) of fecal 16S rRNA gene profiles from control and COVID-19 samples from males (top panels) and female (lower panels) participants at each visit (Visit 1, V1 and Visit 2, V2).

In contrast, PERMANOVA analyses of gut microbiota composition (Jaccard index, Figure 3A,B) and structure (Bray-Curtis index; Figure 4B,D) showed distinctive clustering patterns between control and COVID-19 subjects (*p* < 0.0001 for both indices) at each visit (*p* < 0.01 for both indices). No significant differences were found in beta-diversity between visits. Additionally, the beta-diversity profiles were significantly different between males and females for both Visit 1 and Visit 2 (*p* < 0.05 for both visits). These results suggest that long COVID-19 infection is associated with differences in microbial structure that vary by sex (Figure 4).

Figure 4A–D shows the principal coordinates analysis (PCoA) results.

#### 3.1.1. Gut Microbiome Alterations at the Phylum Level

**Figure 5:**
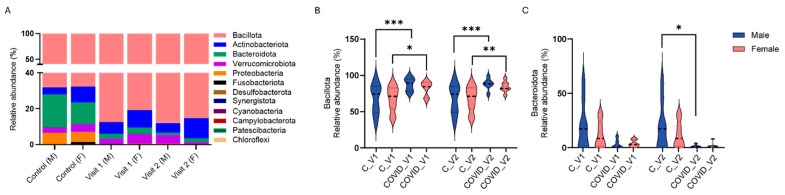
Percent relative abundance of bacterial phyla in male (M) and female (F) controls (C) and in long COVID-19 participants across Visit 1 (V1) and Visit 2 (V2) (**A**). *Bacillota* (**B**) and *Bacteroidota* (**C**) relative abundance in controls and long COVID-19 (Means + SEM, * *p* < 0.05; ** *p* < 0.01, *** *p* < 0.001, Tukey’s multiple comparisons tests).

We assessed the relative abundance of the 12 prevalent bacterial phyla, including *Bacteroidota*, *Bacillota*, Actinobacteriota, Verrucomicrobiota, Proteobacteria, Fusobacteriota, Desulfobacterota, Synergistota, Cyanobacteria, Campylobacterota, Patescibacteria, and Chloroflexi, in male and female controls as well as in Long COVID participants at Visit 1 and Visit 2 (Figure 5A).

Analysis of these bacterial phyla with three-way ANOVA revealed significant effects of visit (F_11,204_ = 241.5, *p* < 0.0001), and group X visit interaction (F_11,132_ = 8.263, *p* < 0.0001), whereas the effects of sex and group or their interactions were not deemed significant. Specifically, the relative abundance of *Bacteroidota* was significantly reduced in males with COVID-19 compared to controls at Visit 2 (*p* < 0.01, Tukey’s test; Figure 5A). While a reduction in *Bacteroidota* was also observed between controls and COVID-19 samples in female participants at both visits (Figure 5C), and between control and COVID-19 males at Visit 1, these differences were not statistically significant. In contrast to reductions in *Bacteroidota*, increases in the phylum *Bacillota* were observed between control and COVID-19 males at both visits (*p* < 0.0001 Tukey’s tests for both visits; Figure 5B) and also between control and COVID-19 females at both visits (*p* < 0.05 for Visit 1, *p* < 0.01 for Visit 2; Tukey’s tests).

#### 3.1.2. Differential Abundance Analysis at the ASV Level

**Figure 6:**
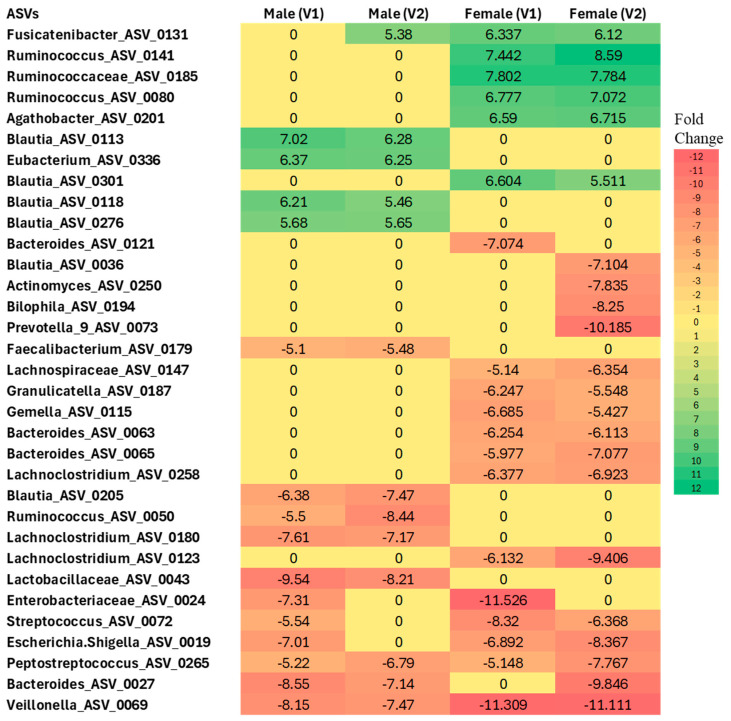
Differential abundance analysis at the ASV level in males and females with Long COVID at Visit 1 (V1) and Visit 2 (V2) compared to controls. Colors indicate the fold change increases (positive numbers) or reductions (negative numbers) relative to sex-matched controls (*p* < 0.005, using Negative Binomial Generalized Linear Model (NEGBIN) implemented in MaAsLin2).

Differential abundance analysis at the ASV level, performed independently for males and females, revealed significant gut microbiome changes in COVID-19 participants compared to sex-matched controls (*p* < 0.05 for all comparisons, NEGBIN implemented in MaAsLin2) (Figure 6).

In females, several taxa were found to be enriched compared to controls, including *Fusicatenibacter* (ASV_0131), *Ruminococcus* (ASV_0141 and ASV_0080), Ruminococcaceae (ASV_0185), *Agathobacter* (ASV_0201), and *Blautia* (ASV_0301) at both Visit 1 and Visit 2. In contrast, several taxa showed a more than 5-fold decrease in abundance in females across visits, such as Lachnospiraceae (ASV_0147), *Granulicatella* (ASV_0187), *Gemella* (ASV_0115), *Bacteroides* (ASV_0027, ASV_0063, ASV_0065), *Lachnoclostridium* (ASV_0258 and ASV_0123), Enterobacteriaceae (ASV_0024), *Streptococcus* (ASV_0072), *Escherichia/Shigella* (ASV_0019), *Peptostreptococcus* (ASV_0265), and *Veillonella* (ASV_0069), when compared to the control group.

Similarly, in males, *Blautia* (ASV_0113, ASV_0118, ASV_0276) and *Eubacterium* (ASV_0336) were found to be enriched at both Visit 1 and Visit 2 relative to sex-matched controls. Conversely, *Blautia* (ASV_0205), *Ruminococcus* (ASV_0050), *Lachnoclostridium* (ASV_0180), Lactobacillaceae (ASV_0043), Enterobacteriaceae (ASV_0024), *Streptococcus* (ASV_0072), *Escherichia/Shigella* (ASV_0019), *Peptostreptococcus* (ASV_0265), *Bacteroides* (ASV_0027), and *Veillonella* (ASV_0069) were significantly less abundant relative to controls.

##### Sex-Specific Microbiome Associations with Inflammatory Markers in Long COVID

Pearson correlation analysis revealed notable sex-specific associations between specific ASVs and blood levels of TNF-α and IP-10 in Long COVID, with distinct patterns observed between males and females across visits (Figure 6).

**Figure 7:**
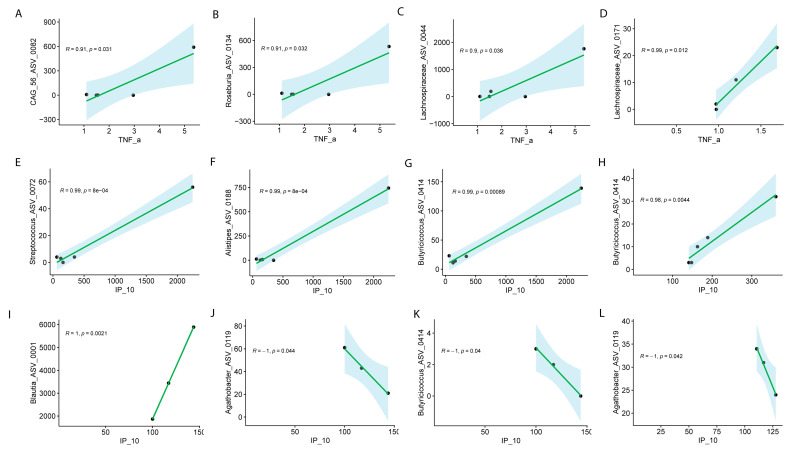
Pearson correlation analysis of ASVs with TNF-α or IP-10 in males and females with Long COVID across visits. Male at Visit 1 (**A**–**C**), female at Visit 1 (**D**) for TNF-α; male at visit 1 (**E**–**G**), male at Visit 2 (**H**), female at Visit 1 (**I**–**K**), female at Visit 2 (**L**) for IP-10. Correlation coefficients (R) and associated *p* values are shown for all significant associations.

At Visit 1, significant associations between specific ASVs and TNF-α levels were observed, with distinct patterns in males and females. In males, strong positive correlations were observed between TNF-α levels and the following ASVs: CAG.56_ASV_0082 (R = 0.91, *p* = 0.031), Roseburia_ASV_0134 (R = 0.91, *p* = 0.032), and Lachnospiraceae_ASV_0044 (R = 0.90, *p* = 0.036) (Figure 7A–D). In females, Lachnospiraceae_ASV_0171 exhibited a similarly strong positive correlation with TNF-α (R = 0.99, *p* = 1.2 × 10^−2^) during Visit 1 (Figure 7D). Neither sex disclosed significant differences for visit 2.

The analysis of specific ASVs with IP-10 (also known as CXCL10) revealed notable sex-specific differences in microbial associations across the visits. In males, several ASVs at Visit 1 exhibited strong positive correlations with IP-10. Streptococcus_ASV_0072 (R = 0.99, *p* = 8.00 × 10^−4^), Alistipes_ASV_0188 (R = 0.99, *p* = 8.00 × 10^−4^), and Butyricicoccus_ASV_0414 (R = 0.99, *p* = 8.90 × 10^−4^) were all significantly positively correlated with IP-10 at Visit 1 (Figure 7E–G), indicating a strong relationship between these microbial taxa and immune response markers in males. Additionally, *Butyricicoccus*_ASV_0414 continued to show a significant positive correlation with IP-10 at Visit 2 (R = 0.98, *p* = 0.0044) (Figure 7H–K). In females, the correlations were more varied. At Visit 1, *Blautia*_ASV_0011 exhibited a perfect positive correlation with IP-10 (R = 1, *p* = 2.10 × 10^−3^), while *Butyricicoccus*_ASV_0414 (R = −1, *p* = 4.00 × 10^−2^) and *Agathobacter*_ASV_0119 (R = −1, *p* = 4.20 × 10^−2^) showed strong negative correlations with IP-10 (Figure 7L). Interestingly, *Agathobacter*_ASV_0119 continued to show a significant negative correlation with IP-10 at Visit 2 (R = −1, *p* = 0.042) (Figure 7L). These results highlight the complex, sex-specific interactions between the microbiome and immune modulators, such as IP-10, suggesting that different microbial communities may influence immune responses in males and females differently in Long COVID. We did not collect menstrual data, but depending on age, this might have had an influence of symptomatology.

##### Inference of Functional Pathways Associated with COVID-19

**Figure 8:**
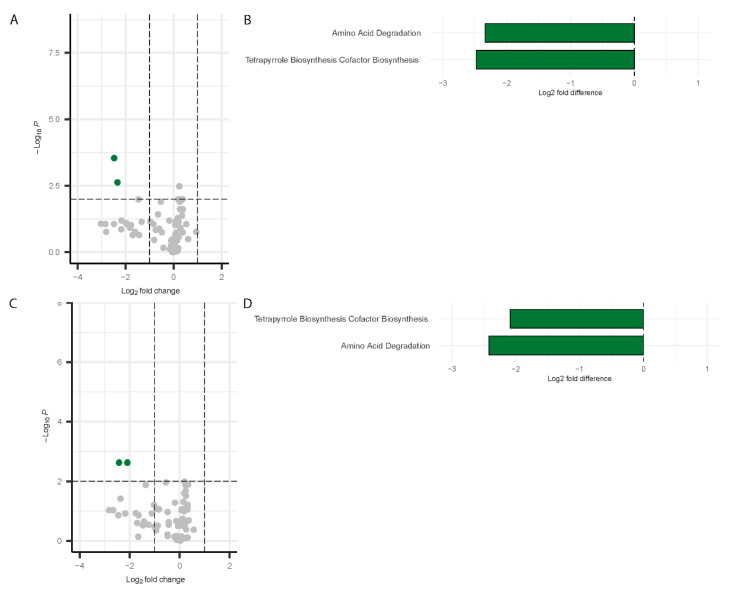
Differentially abundant pathways in males. Volcano plots showing differentially abundant pathways for males (Visit 1, (**A**); Visit 2, (**C**)) with bar plots depicting the differentially abundant pathways (Visit 1, (**B**); Visit 2, (**D**)). Green dots represent the significantly downregulated pathways whereas grey dots represent the unchanged pathways relative to controls.

Differential pathway analysis on the microbiome data using PICRUSt2 [52] detected significant changes in pathway abundance for both males and females during Visit 1 and Visit 2. The results are visualized using a volcano plot and a bar chart. For males at Visit 1, two pathways were significantly downregulated by more than two-fold change: Amino acid degradation and tetrapyrrole biosynthesis (Figure 8A,B). These same pathways remained downregulated at Visit 2 in males (Figure 8C,D).

**Figure 9:**
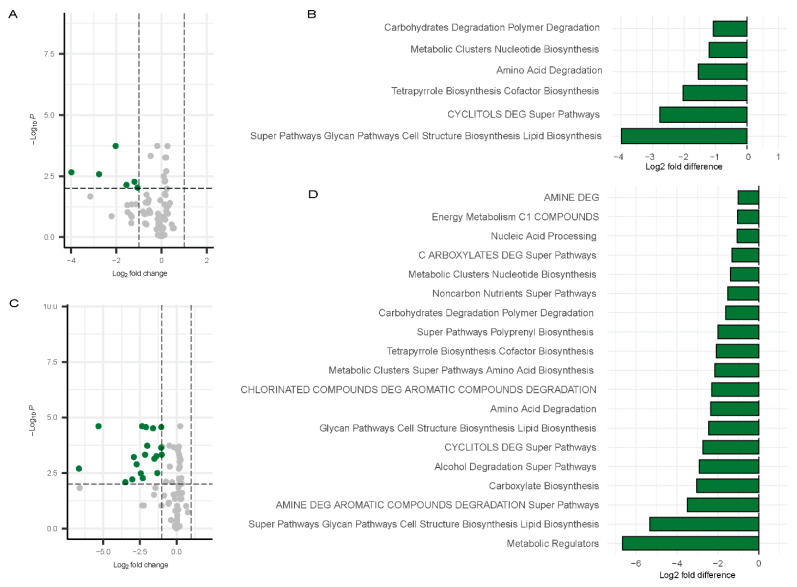
Differentially abundant pathways in females. Volcano plots showing differentially abundant pathways for males (Visit 1, (**A**); Visit 2, (**C**)) with bar plots depicting the differentially abundant pathways (Visit 1, (**B**); Visit 2, (**D**)) compared to controls. Green dots represent the significantly downregulated pathways whereas grey dots represent the unchanged pathways relative to controls.

In contrast, a broader set of pathways [53] exhibited significant downregulation in females at Visit 1. Figure 9A,B) shows data relative to controls. These included carbohydrates degradation polymer degradation, metabolic clusters nucleotide biosynthesis, amino acid degradation, tetrapyrrole biosynthesis cofactor biosynthesis, CYCLITOLS DEG super pathways, and super pathways glycan pathways cell structure biosynthesis lipid biosynthesis (Figure 9C,D). These pathways were also depicted in the bar chart to highlight the fold changes (Figure 9A,B). At Visit 2 in females, a broader range of pathways were downregulated, with the following showing at least a three-fold change: Metabolic Regulators, Super Pathways, Glycan Pathways, Cell Structure Biosynthesis, Lipid Biosynthesis, AMINE DEG, Carboxylate Biosynthesis, and Alcohol Degradation Super Pathways. (Figure 9C,D). The greater downregulation of these pathways in females at Visit 2 suggests a more pronounced shift in microbial function over time in females.

Overall, these results highlight notable sex-specific variations in the microbial metabolic pathways affected by Long COVID, with females exhibiting more downregulated pathways than males.

##### Correlation Between Gut Microbiome and Inflammatory Markers

MaAsLin2 was used to examine the association between specific ASVs and levels of TNF-α and IP-10. The analysis was performed using ASV abundance profiles, group (COVID-19 vs. control), sex, and visit number as input data. The model included fixed effects for group, sex, visit, and TNF-α or IP-10 levels, while subject ID was included as a random effect to account for intra-subject variability. A negative binomial regression model (NEGBIN) was applied to account for overdispersion in the count data. To ensure robust results, a minimum prevalence of 33% across samples was required, and ASVs with a q-value of <0.05 were considered significantly associated with TNF-α or IP-10 levels. Following the identification of significant ASVs, analyses with Pearson correlation were performed to further explore the relationship between inflammatory marker levels and the relative abundance of these ASVs stratifying by sex and visit.

##### Inference of Functional Genes and Pathways

Functional pathway occurrence was predicted based on 16S rRNA gene sequences using the Phylogenetic Investigation of Communities by Reconstruction of Unobserved States (PICRUSt2) software package version 2.5.2 [52]. This software infers the presence of functional pathways using marker gene sequences obtained from 16S rRNA sequencing data. MetaCyc ontology predictions were employed for classifying metabolic pathways [53]. Differential abundance testing of inferred pathways was conducted on count data using the R package MaAsLin2 (v 1.14.1). NEGBIN was utilized within MaAsLin2 to assess the abundance of inferred pathways related to experimental conditions. The model accounted for fixed effects for both participant sex and visit.

##### Serum Cytokines

We analyzed 19 cytokines in patients with COVID or PASC. The cytokine properties are tabulated below, in the footnotes of Table 3.

We also contrasted the disease states of COVID and PASC for correlations between different cytokines, as summarized in Table 4.

**Figure 10:**
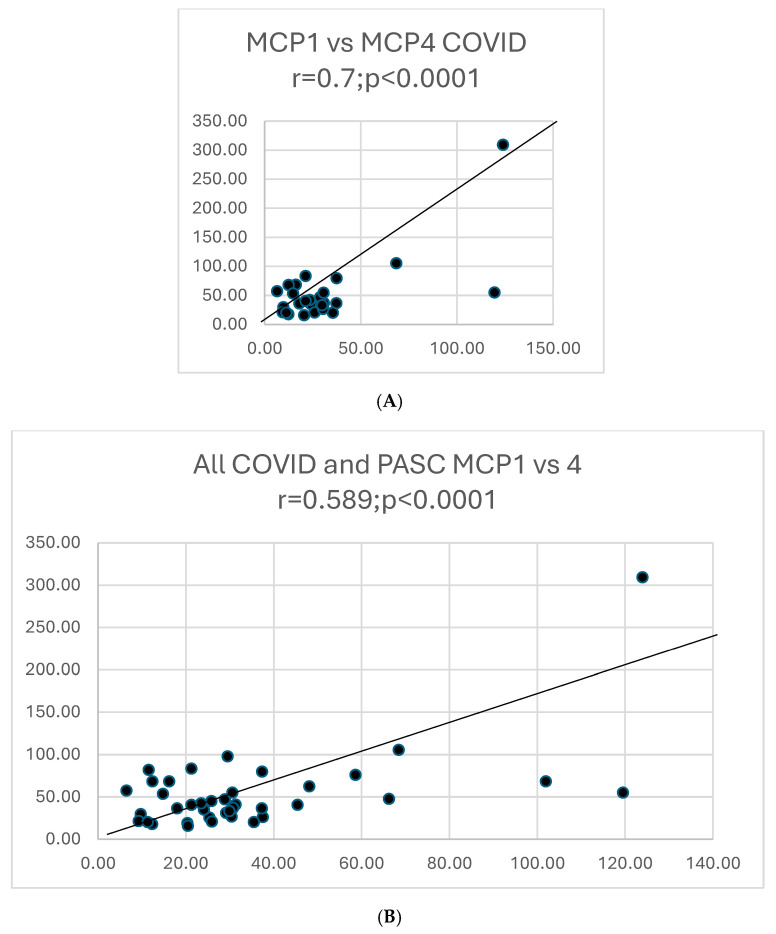

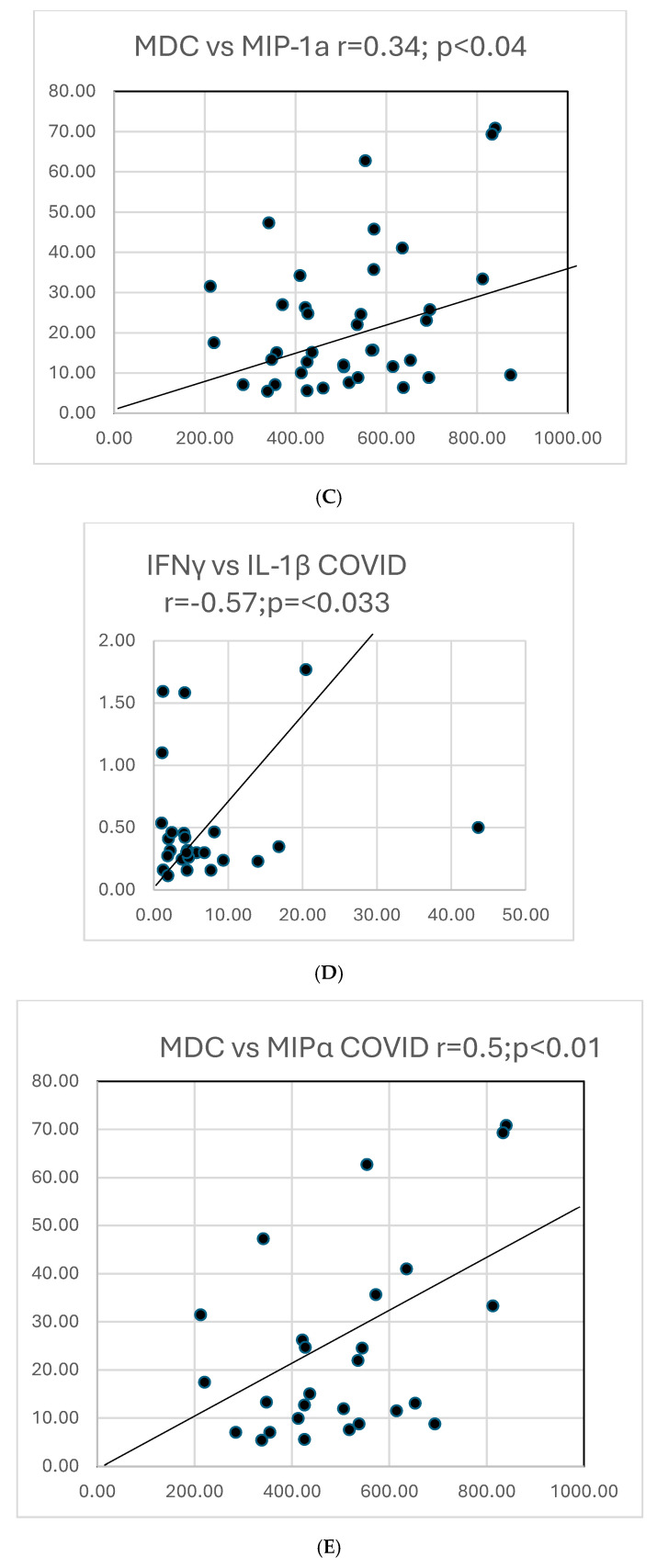
**(A**). A scattergram shows the correlation of two cytokines MCP1 versus MCP4 in COVID. The results correlate well. (**B**). A scattergram shows the correlation of MCP1 vs. MCP4 in all COVID-19 and PASC patients. There is a high degree of correlation MCP in these 2 groups. (**C**). A scattergram shows the correlation of MDC vs. MIP-1a. The correlation is significant for a direct correlation between the two cytokines. (**D**). A scattergram shows the correlation of IFNγ vs. IL-1β. A direct relationship is seen between these two cytokines. (**E**). A scattergram shows the correlation of MDC vs. MIPα in COVID19 patients. We also contrasted proportions of positivity of cytokines in COVID and PASC.

Graphs showing selected correlations are presented below (Figure 10).

**Figure 11:**
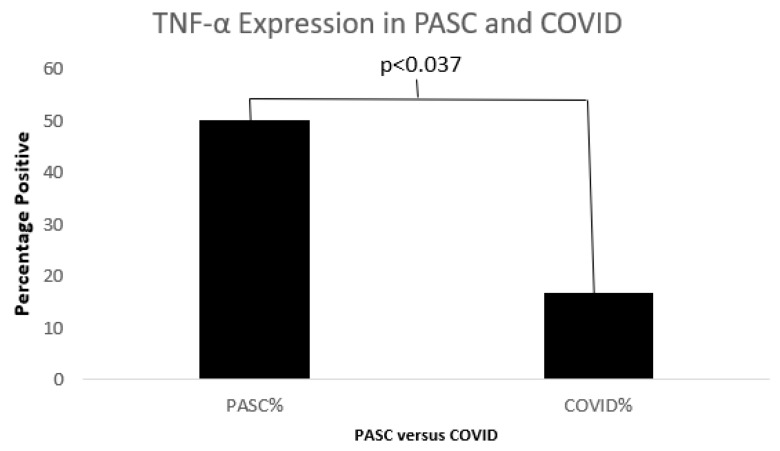
A bar diagram shows a significant difference in TNFα between COVID19 and PASC.

**Figure 12:**
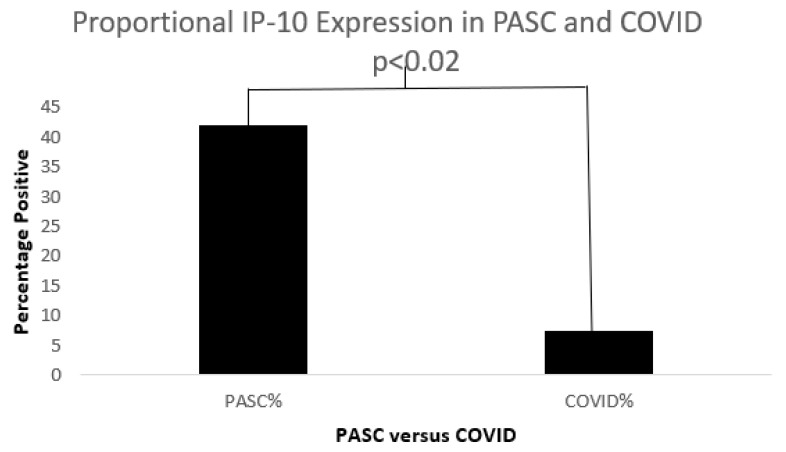
A bar diagram shows a significant IP-10 expression difference in both COVD-19 and PASC patients.

**Figure 13:**
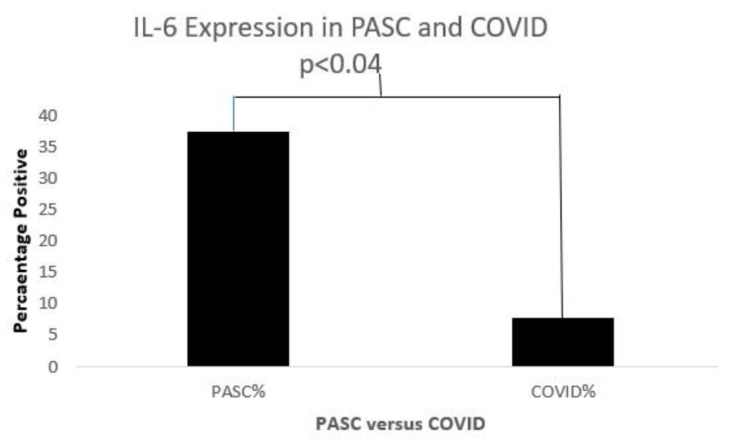
A bar diagram shows a significant IL-6 expression difference in both COVD-19 and PASC patients.

Figure 11, Figure 12 and Figure 13 below show cytokine expression in PASC and COVID-19.

##### Database Correlates with Chronic Fatigue

Practically speaking, worldwide, there are many individuals with these precedent syndromes who are disabled and are supported financially despite the less than full understanding of the pathophysiology. This is of practical significance to those with Long COVID who could be afforded the same benefits. Older and more novel innovation into cognitive dysfunction in this patient population will also be highlighted. In addition, we describe the potential for animal models which would allow us to better understand both the acute and chronic COVID-19 etiologies and likely provide similar insights into CFS/ME, advancing both entities. With the focus on widespread human misery, release and exposure of societal ills and divisiveness that the COVID-19 Pandora Pandemic has caused and continues to cause, we end with a fitting epilogue from a poet of a country that espoused renaissance societal, ideological foundations to equalize the social strata, which remains applicable to the COVID era.

Overlaps are expected and little comparative biomarker data have accrued to guide this short review to allow current research discovery to beach onto unexpected shores. We have tabulated similarities and difference for the reader’s interest along with relative citations which will hopefully reignite interest and lead to cogent research efforts.

One of the most important aspects of COVID-CoV-2 SARS infection is the phenomenon of “Long COVID”. This poorly understood entity has posed an existential threat to well-being even when this pandemic, which has caused so many health and socio-economic fissures to disrupt the lives of humankind (hence Pandora Pandemic), appears to be slowing down in some areas of the world, only to reemerge unexpectedly. The difficulty of defining this “syndrome” have been exhaustively documented [1].

**Figure 14:**
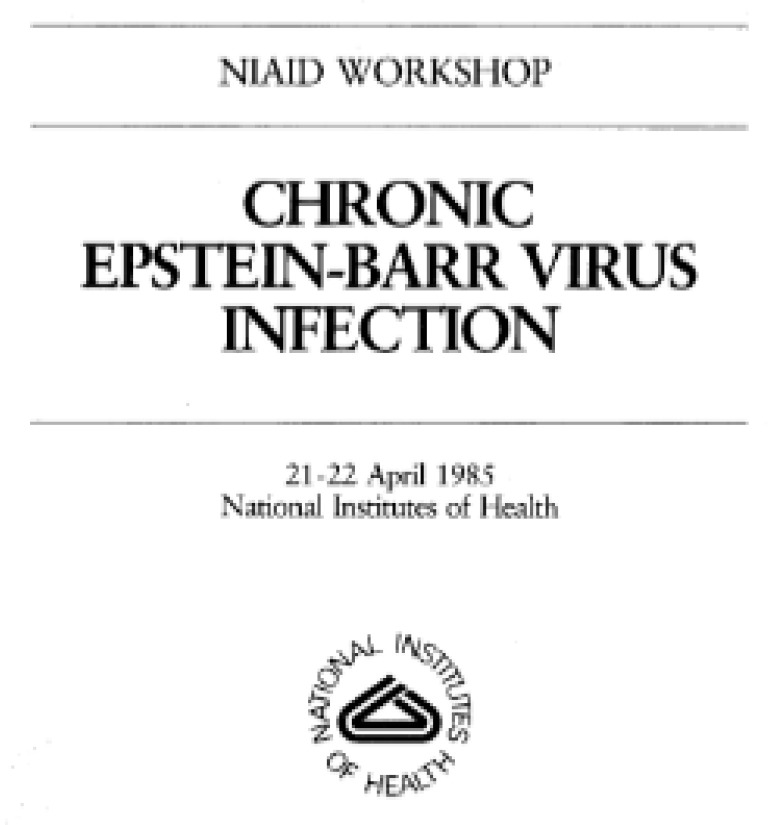
Face page of the NIAID Chronic EBV Infection Conference Program in 1985.

Next year will mark 40 years (see Figure 14 below) of inability to adequately define or effectively treat this syndrome, which hopefully will not be the case with Long COVID. For this reason, we equate CFS/ME and Long COVID within this review.

Indeed, rintatolimod [8] is an as yet unapproved drug that is touted to increase OAS levels without helicase activation; it is available, but there are further hurdles to overcome. Undoubtedly, much work needs to be completed before this approach becomes a much-needed reality. Table 5 shows that the approaches to these syndromes may intersect, and that OAS synthetase should be estimated in these patients. This is not mere speculation, as it appears that an OAS isoform originating in Neanderthals reduces both susceptibility and severity in Europeans [7]. If this biomarker is confirmed to be elevated in these patients, effective biologics could be designed to reduce levels, assuming that these levels are indeed the salient features of chronic EBV, CFS/ME, and Long COVID.

##### Cognitive Testing in Long COVID

There is a statement that “hospitals are bad for sick people,” and there is some truth to it. Cognitive disability may be part of a pre-existing condition or may develop due to a new disease process, and sometimes, teasing this conundrum into its relevant components can be quite challenging. We encountered this in our early experience in determining cognitive disability between two groups of hospitalized older adult populations: one group with hip fractures, and one admitted for cerebrovascular accident (CVA). Interestingly, we found that the cognitive disability in the former group was significantly greater than the latter [23]. This is not surprising given that the falls were likely the result of a disability of cognitive spatial appreciation resulting in misjudgments in ambulation, which led to serious falls and musculoskeletal trauma. Almost 40 years later, in the COVID-19 pandemic, a resourceful group of British scientists sought to conduct the same cognitive testing on COVID19 patients [24]. By partnering with a major media outlet, they were able to use an app and obtain data ascertaining multiple facets of cognitive impairment. They were careful not to allow selection bias into their dataset, but offered this app as a means to allow the individual to ascertain their best level of cognitive function; they did not include COVID-19 in their promotional announcements. About 83,000 citizens completed the cognitive exercise. The responses were self-reported, and no confirmatory evidence was sought. Although these are serious pitfalls in any scientific undertaking of such enormous scope, the investigators were able to glean a number of important outcomes.

The first inescapable reality is that COVID-19 appears to be associated with cognitive disability at a level greater than CVA patients or patients with a learning disability (SD 0.47, which equates to a 7-point drop in IQ standard testing). Perhaps not surprisingly, those with severe respiratory symptoms were at highest risk but, ominously, those with milder severity also suffered from the same cognition deficits that have also been encountered in other Long COVID reports, unexplained by the usual demographic [24] predispositions (gender, age, and obesity). These deficits remained on a greater scale than those seen with other viral diseases. The cognitive decline was greater than that seen in a 10-year span of natural age-related decline, and this might be clinically significant. The magnitude of the reduction in executive higher-order cognition was found to be similar to that of pre-pandemic ARDS patients, which could persist at a five-year follow-up mark.

A unifying factor in chronic COVID-19 prevention, Long COVID, and CFS may be the myocardium; this was highlighted in Part 2 as a potential explanation of vaccination-related myocarditis. Dr. A. Martin Lerner, who was a CFS researcher for 25 years and infectious disease specialist, and who was diagnosed with CFS in 1988, felt that the cause of CFS was infection in the heart by the EBV (URL: https://today.wayne.edu/medicine/news/2015/10/07/dr-a-martin-lerner-longtime-chief-of-infectious-diseases-dies-at-86-29387, accessed on 15 May 2025).

These findings will need to be confirmed with more direct testing, but this study confirms the cognitive deficit described above in the fatigue syndrome and is part of that constellation of neuromuscular and endocrinal dysfunction. He and his group also implicated CMV in the paradigm including a 3rd subset, i.e., coinfection with both CMV or EBV [25]. They bemoaned the lack of consistent markers for CFS but accepted positive EBV or CMV IgM as evidence for primary or reactive infection and innate immune activation of {2-5′}-Oligo-A-Synthetase or its dependent ribonuclease I moiety. They concluded that cardiac involvement was a unique feature, proven by myocardial biopsies, and that the observed incomplete viral replication leads to a non-inflammatory progressive myocytic apoptosis, with attendant EKG changes on monitoring and chronic fatigue. They did not advocate for myocardial biopsies after two patients in their series developed hemopericardia. They further suggested that interventional studies with anti-viral agents proved somewhat successful when using their Energy Index Point Score and established end-point criteria and suggested protocols.

In striving to understand the connection between Long COVID and other post-viral syndromes and the underlying immune imbalance, we turn to our experience with US veterans. These veterans do experience the above syndromes, albeit rarely but significantly, when introducing the element of stress (overseas deployment); this is in contrast with those who have not been deployed [26]. It appears that the CFS variant is almost 18 times more prevalent in terms of odds ratio (40.6 versus 2.32) in those who have been deployed, and therefore more significant. The confidence intervals given are 10.2–16.1 and 1.02–5.27 comparing CFS and Fibromyalgia, and the difference between the deployed and the undeployed is 1.6 versus 2.0% and 1.2 versus 1.6%, respectively. In order to clarify potential underlying disorders, we identified long-term follow-up data on 13 patients with fatigue who were designated in the problem list of the computerized patient record system (CPRS) as having ME/CFS or chronic fatigue. For a control group, we used Group 2 patients (see Part 1) who were suspected of having a viral infection such as COVID-19 or influenza but tested PCR-negative for any viral infection, with an additional seven patients found on follow-up (*n* = 53; Part 1, Table 1). For susceptibility data, we used the data from the 1542 patients of the diabetes analysis set (see Part 2).

**Figure 15:**
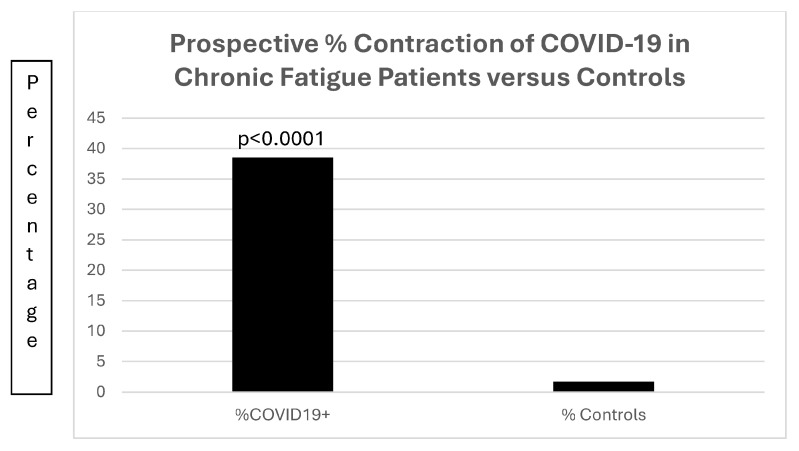
A bar diagram contrasting the preponderance of prospective COVID-19 infections in patients with chronic fatigue.

Of much interest is the increased susceptibility (Figure 15) of the 13 patients with chronic fatigue; in that group, 5 of the 13 (38.5%) patients contracted COVID-19, compared to 26 of 1542 (1.7%) of patients with diabetes (OR22.43[7.45–67.50]; *p* < 0.0001).

Figure 15 is a bar diagram depicting the significant difference in patients with chronic fatigue contracting COVID-19 as compared to a control population of patients with diabetes mellitus.

This strongly suggests that lifestyle/immunity factors are strongly involved in the fatigue group. To analyze these possibilities, demographics are summarized in Table 6.

Table 6 shows that demographics of patients with chronic fatigue and controls are similar, with no predisposition to chronic infections such as a history of gastric helicobacter pylori infection or conditions with the potential to cause inflammation, such as smoking and alcohol consumption. No differences in hard endpoints such as survival were seen, and the only significant difference was the expected tendency to be overweight in the controls, all of whom had diabetes mellitus.

**Figure 16:**
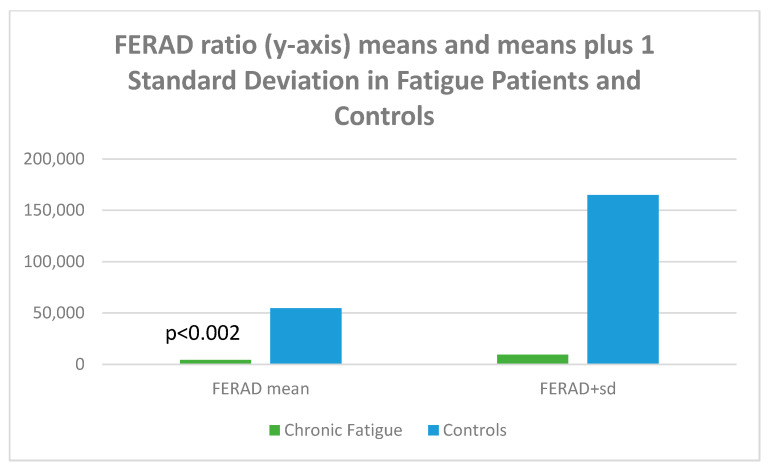
A bar diagram depicting the differences of FERAD ratio in fatigue and control patients.

Given the accuracy of the FERAD ratio (blood ferritin/stool ELISA OD-background of Adnab-9 monoclonal binding) in predicting susceptibility and severity (the latter a biomarker for Long COVID), we looked at aspects for all p87 components. The FERAD results are depicted in Figure 16.

Figure 16 is a bar diagram showing that the mean FERAD ratio difference is statistically significant for patients with chronic fatigue as compared to controls, with the mean on the left and the means plus 1 standard deviation of FERAD ratio on the right; the figure therefore depicts the variation of the data.

**Figure 17:**
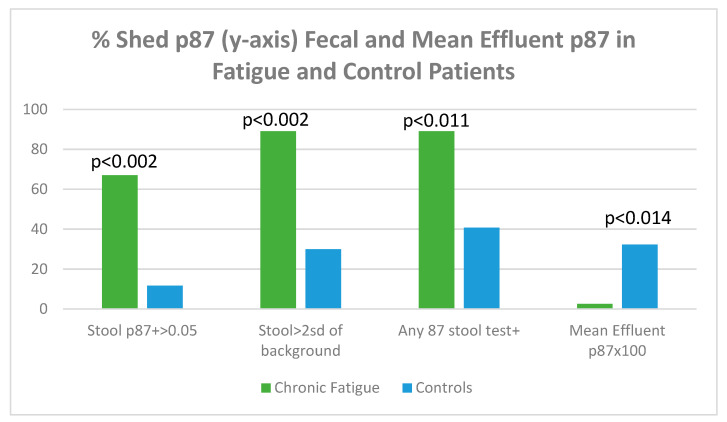
A bar diagram showing levels of shed p87 in the stools and effluent in fatigue and control patients.

Figure 17 displays shed p87 antigen in stool using binomial (OD > 0.05 over background) or tests > 2 standard deviations from an entire set of background readings on the ELISA plate.

Figure 17 shows that, regardless of how p87 positivity is expressed, all fecal shed p87 is significantly higher in the chronic fatigue group of patients. The p87 in effluent, magnified by 100× for clarity shows a paradoxical effect of significantly reduced effluent p87 estimations. The shed p87 in the effluent was less in the fatigue patients 0.026 ± 0.033 (OD minus background) versus control effluent samples 0.323 ± 0.522 by the Student’s t-test (*p* < 0.014). These effluent findings are similar to the significant inverse relationship (*p* < 0.12) seen between effluent and stool p87 estimations [Vide Infra].

Regarding retained p87 by semiquantitative immunohistochemistry, no p87 appears to have been detected in the fatigue patients’ cecum, ascending, or sigmoid colon, but the sample was small. In contrast, in the control patient, the p87 in the detectable cecum was 0.417 ± 0.575 versus zero; ascending colon was 0.222 ± 0.392 versus zero; and sigmoid was 0.194 ± 0.389 versus zero, suggesting loss of antigen or Paneth cell function. This may also explain why more shed antigen was found in the stool.

For completeness, given that this fatigue group may have other deficits compared to controls, we looked at a variety of other parameters, such as inflammatory, organ functionality, and medications. These are summarized in Table 7.

There were no significant differences in taking aspirin or other NSAIDs (*p* = 1), but there were trends to lower iron saturation levels in patients with fatigue (15.81 ± 12.41 versus 25.30 ± 14.75 in controls, *p* = 0.076) and with vitamin D levels (25.43 ± 12.77 versus 16.99 ± 8.45 in controls; *p* = 0.091). The issue of tonsillectomy is intriguing, as some researchers describe a tendency for higher fever [28], and others conclude that there is no increased risk of contracting COVID-19 or having higher severity [29].

Long COVID papers (*n* = 61) were reviewed. All papers considered patients with sustained symptoms >28 days after the initial infection, with varying times of follow-up. Symptoms appeared to decrease with time. Only one study included a comparison group of patients (healthy controls), which is a serious shortcoming and a methodological flaw. What results is a conglomeration of banal statistics with the occasional focus on one system or another depending on the expertise of the author/s, but there were a few significant conclusions and some conflicting information (Table 7). Many studies contained meta-analysis (MA) data, which can introduce bias, and other papers which used MA data had as few as five patients with no control group. Many, as anticipated, decried the lack of certainty as to the exact nature of Long COVID at this stage of our understanding, and so we avoided promoting long lists of symptoms and signs other than those of the enumerated studies. Others draw a parallel, at a great cognitive distance, to other post viral syndromes, persistent disease states, and longitudinal emergence of extreme stressor hospital environments, such as post intensive care-like syndromes, which remain to be elucidated.

**Figure 18:**
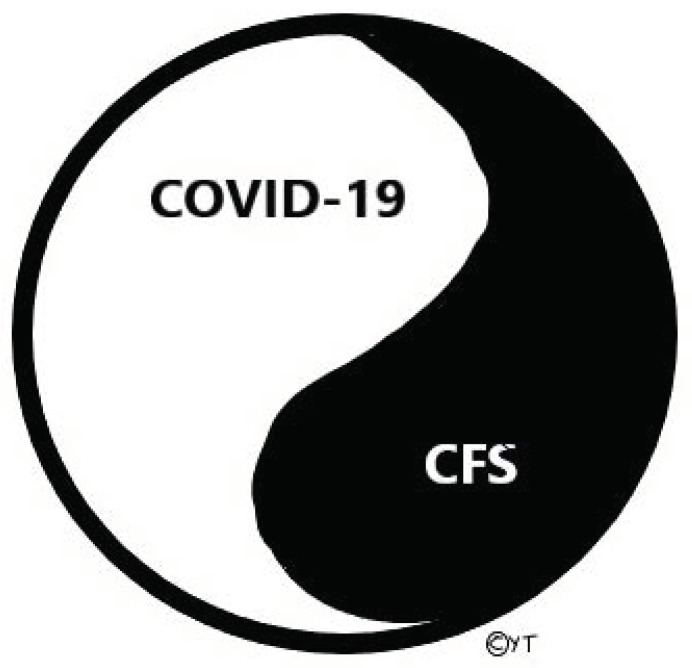
Traditional depiction of the Yin and Yang interactions adapted to show the likely co-relationships of chronic post-viral syndromes.

Conceptionally, what we and others have perceived is a Yin and Yang spatial relationship in which chronic fatigue syndromes and Long COVID states co-generate into a continuous replicating cycle of causation, the details of which can be appreciated in Figure 18.

Selected publications that may offer insight into the features and possible underlying mechanisms, and even a final pathway of causation with other fatigue and chronic organ syndromes, have been summarized in Table 8.

## 4. Discussion

There is no doubt that this body of work is extensive, involving a variety of disciplines brought to bear on the conundrum of chronic fatiguing illnesses with which we have been involved since our seminal paper in 1982. While our primary focus is on Long COVID, and we have devoted two sections of this paper addressing the microbiome and cytokines, we have also considered historical post-viral syndromes and chronic fatiguing illnesses that still evade scientific explanation, with hundreds of thousands of individuals around the world suffering from these maladies that affect almost every aspect of their lives. We do not purport the notion that the triumvirate foundation espoused in this paper is necessarily the cause of these illnesses, but applying them may be useful in terms of gaining a better understanding of the etiology, leading to interventions that may alleviate these maladies. There were also no differences seen in males and females at Visit 2, which points to similar reactions to PASC which persist with follow-up.

While reductions in microbial alpha diversity have been described in individuals with COVID-19 (Horvath et al., 2024) [73], we did not find any significant changes in this study. This could be explained by differences in the severity of the infection and time post- COVID-19. Notably, reports of gut microbiome effects of COVID-19 by sex are scarce. Similarly, differences in the composition of the gut microbiome after infection with COVID-19 have been documented, including decreases in Ruminococcaceae (Horvath et al., 2024) [73]. In this study, COVID-19 resulted in decreases in the abundance of the phylum *Bacillota* in both males and females, whereas *Bacteroidota* was only significantly reduced in males. This not only indicates a profound dysbiosis associated with long COVID-19 infection, but also highlights sex-dependent outcomes.

Associations between specific microbial ASVs and blood levels of TNF-α and IP-10 in long COVID-19 were also sex-dependent. In males, several ASVs were positively correlated with both TNF-α and IP-10, while females exhibited some ASVs that were positively and negatively correlated with these immune markers. This suggests that microbial communities may influence immune responses differently in males and females with long COVID-19.

Results from the differential pathway analysis on the microbiome data with PIC-RUSt2 showed a downregulation of the Amino acid degradation and Tetrapyrrole bio-synthesis pathways in males at both visits. This could reflect metabolic slowing or re-programming seen in chronic conditions like ME/CFS.

In females, the downregulation of carbohydrate degradation, nucleotide biosynthesis, amino acid degradation, tetrapyrrole biosynthesis, and lipid biosynthesis may reflect a system-wide metabolic reprogramming in PASC and ME/CFS.

This study has several limitations, including the relatively small sample size, which was further reduced by stratifying participants by visit and sex. Additionally, variability in the timing between COVID-19 diagnosis and the second study visit may have influenced the results. However, these limitations are balanced by the fact that data on both gut microbiome alterations and blood cytokine profiles in post-COVID-19 individuals remain scarce, highlighting the value of the findings.

To complete the coronavirus picture, we would be remiss not to explore the natural world from whence this family of viruses emerged and became scientifically detectable in the 1930s. Since we have some experience in monkeys and mice, we should like to introduce an outbreak of coronavirus apparently distinct from SARS-CoV-2 [36]. Even in those relatively early days, the authors appreciated the fact that these viruses had the largest RNA genome of all the known RNA viruses. This may explain the versatility and rapid mutation rates that may have produced a number of mutated clades.

One of the surprising findings from the above animal study of a coronavirus outbreak is an historic electron micrograph of a coronavirus devoid of its nucleocapsid. This was taken from an article published in 1985 [37], but it is not known whether this particle was infectious. While wasting diarrhea was the dominant feature of the described outbreak in animals, a saddleback tamarin (Saguinus fuscicollis) had bronchopneumonia and nephritis without diarrhea, and while coronavirus was identified in the stool, no further characterization was completed. This is disturbing, as the disease was fundamentally different in the tamarin than in the other animals, and it had pathologic features more in line with the SARS group of coronaviruses. This does suggest, however, that this species of tamarin may be an appropriate non-human primate model for COVID-19, should the need arise. We have shown that these tamarins (mainly cotton top and common marmosets), express many antigens, including p87, CEACAM-1 (mouse hepatitis coronavirus ligand), and p38 MAPKs, amongst others [38].

A recent review of potential animal models for COVID-19 [39] points out that, unfortunately, mouse ACE2 receptors do not bind effectively to the M spike protein, and unless the mouse can be genetically engineered with humanized ACE2 (hACE2) as has studied for CNS infectivity, it is not an option for a convenient animal model. Ferrets, minks, non-human primates (rhesus macaques, African Green monkeys), cats, Syrian hamsters, dogs, and bats could all offer information on the various stages of COVID-19 infection and transmission. Most important is the understanding of the immune response to the virus. Animals that are less suitable include pigs and poultry (ducks and chickens), which are not susceptible to the virus. Cell lines, both human and canine (MDCK), may be useful. From our point of view, animals that lack Paneth cells, such as cats and dogs, where p87 is not detectable (unpublished data) or elaborated, would be useful for infection experiments, but would not mimic human infections or gut immune cells. Rats were not mentioned in this review, but they are susceptible to coronavirus strains and have been used in studies that test for anti-COVID-19 medicinal delivery [40]. Interestingly, a recent review was related to the aforementioned delivery of ivermectin, and this agent has also been touted to treat COVID-19 [41]. The review also included publications that advocated using this agent to treat acute and chronic illnesses, including the short and long forms of COVID-19 and even CFS, but the primary review cited those publications and considered them skeptically, calling them low grade. As a link to Part 2, it has been postulated that vaccines may exacerbate the severity of COVID-19 pulmonary disease in which the cells express a Fc receptor [42], so this must also be considered.

While we have endeavored to present a comprehensive and cohesive interpretation of the reaction of the innate immune system to viruses such as SARS-CoV-2, EBV, and other herpes and coronaviruses, there are others that are related (such a cytomegalovirus) and unrelated, yet to be discovered. In this regard, the major pitfall of this paper is its reliance on the above viruses to garner most of the data it uses. Efforts should be made to expand the viral repertoire to include other common viruses. The other shortcoming is that the number of PASC patients researched is rather small, and larger numbers will be needed to expand this experience. The strength of the paper is its reliance on the microbiome to make cogent analysis leading to a strengthening of metabolome data from both cytokine and microbiome data.

There is no doubt that these syndromes exist. The etiologies may vary, but manifestations are so broad in scope that there is much overlap. Consequently, there is a need for further research to better delineate these syndromes and identify therapeutic interventions, as we have elaborated. The use of animal models for these syndromes is in its infancy, but it is clear that COVID-19 is a zoonotic disease, and defensive immunologic strategies may be available to modify the reaction of the innate immune system to alleviate chronic symptoms as we have outlined, and grounds for feasibility have been presented. Despite our optimistic view, the reality is that this will take time, and the verses below do embody the current frustration with this Pandora Pandemic. The topics described in this section are broad, and the disciplines involved are varied; they span many of the National Institutes here in the US. We believe that a new institute should be created, which might be called the National Institute of Pandemics. This would incorporate all the disciplines that do not overlap but would provide a unique focus on acute and chronic disease, something that is missing at this juncture, and which would speak with one assuring voice.

We stand on the shoulders of giants to gain a better perspective, including George and Eva Klein of the Koninglike Karolinska Institutet who dealt with Chronic Mononucleosis and identified Epstein Barr virus (EBV) as a potential cause; Martin Lerner of Wayne State University, who followed this path despite battling the same disease, and confirmed anti-EBV IgM persistence, as we originally reported; Werner and Gertrude Henle of the Children’s Hospital of Philadelphia, who were also active in refining EBV serology; Alexis Shelokov of the Scripps Institute, Government Vaccination Branch, who discovered the Islandic chronic disease syndrome, amongst others; and those who participated in the 1984 NIAID (National Institutes of Allergy and Infectious Disease) conference, many of whom have gone on to become prominent contributors to this field. Building on this firm foundation, we offer our insights into the microbiome changes and activity of the cytokine system. There will be numerous other like-actors that cause these disease syndromes, and we hope that our contribution can be a firm foundation which can be used by those who come after us to build a clear understanding of the mechanisms and introduce medical interventions to free mankind of this scourge.

Epilogue:
And out came the grief and woe
We won‘t ever be rid of,
For heaven had hidden
That in the jar.
Isaac de Benserade, French Poet 1676, *Métamorphoses d’Ovide*

## 5. Conclusions

We look forward to disseminating the knowledge we present in this paper particularly for the authors who perfected the PASC determination, and believe that, in the near future, these syndromes will be a thing of the past although, we must always stand vigilant. The next steps are to perfect the medications that can be directed against the immune players to ameliorate the suffering poses by PASC and challenge young investigators to expand this into new vistas with a unique perspicacity to address the vicissitudes and idiosyncrasies of the onslaught of disease to which humankind is prone.

## Figures and Tables

**Table 1:** Demographics of patients whose data were retrieved from the biorepository.

Patient Number	Age	Sex	Ethnicity	Employed:Unemployed/R*
*n* = 28^	47.1 ± 15.7 yrs	M:F 17:11	Cau:24; His:3; As:1	17:1/5

R*—retired; yrs—years; M—male; F—Female; Cau—Caucasian; His—Hispanic; As—Asian; ^—BMI 28.47 ± 5.7.

**Table 2:** Morbidity and exposures.

Smoker Status	Other Household	Hospitalized	Intubated	Received Oxygen
1 smoker; 27 none	Occupants 2.4 ± 1.4	10+14−42%	2+22−12.5%	9+15−37.5%; (average saturation: 9.7 ± 26.5%)
Other Measures/Conditions	3+21−12.5% received convalescent serum	1 pt expired 2 received vaccination	5:19 diabetes 21%	1 Expired 0.04%

**Table 3:** Summary of the different chemokines used and the clustering of downstream effects.

Designation	Function	Disease Role	Conjunction	DX	SubFam	Ref.
Eotaxin	Eosinophil rec.	Pancreatic	HGF; MCP-1; CXCL10	s.86 sp92	MCP-1	[54,55]
Eotaxin-3	Eo.Chemotactic	Unknown	MIF-4α; TSC-1; IL-4	Unknown	CCL26	[56]
IP-10	Growth, Apop	Infections	IFN γ-induced pr-	Severity Pre.	CXCL10	[57]
IL-8	Gprotein-couple	Neutro Attract	Macrophages	Inflammation	CXCL8	[58]
MCP-1	Monocyte Attrac	IFNγ induct.	CXCR3; predict: COVID	Inflammation	CCL2	[59]
MCP-4	Allergic Reaction	Asthma	TNFα&IL1β; IFN-γEo	Inflammation	CC chem	[60]
MDC	Amplication Type2	Immunobiol	IL-4; IL13; IFN, CCR4	Regulation	CCR4 chem	[61]
MIP-1α	Divergent signal	Inhibition	CCR5; M-tropic HIV	Inhibition HIV	CCL3	[62]
MIP-1β	Related to above	Immunity	CCR5; M-tropic HIV	Immunity	CCL4	[63]
TARC	Evade immunity	Melanoma	Dendritic cells	Improve Mel	CCL17	[64]
IFN-γ	Viral Inhibition	Viruses	Lymphocytes	Type II IFN	Dimerized	[65]
IL-1β	COX2 induction	Inflammation	Dendritic/monocytes	Autoimmune	slanDC	[66]
IL-10	Inhibit Inflammation	Jak; TYKinase	T-reg cells; IL-10R’s	Inhibitor DC	Class 2	[67]
IL-12p70/p40	T to Th1, NK cells	Stim IFN-γTNFα	Dendritic cells prod	Diverse functions	Het-dimeric	[68]
IL-2	Infection reponse	wbc regulation	Leuco-& lymphocytes	Increase H&T cells	interleukin	[69]
IL-4	IgE Class switching	Allergies	Differentiation Th cells	Mast cell stim	interleukin	[70]
IL-6	Pro-inflammatory	COVID-19 prog	Activates IL-1ra&IL-10	osteoblasts	interleukin	[71]
TNF-α	Pro-Inflammation	Immune-Stim	Active Tri-homotrimers	Macrophages	T-Membrane	[72]

DX—diagnosis; SubFam—sub-family; Ref.—reference; rec.—recruitment; HGF—hepatocyte growth factor; MCP-1—monocyte recruitment protein-1; s.—sensitivity; sp—specificity; TSC—thymic stoma chemokine-1; IL-4—interleukin-4; Eo—eotaxin; IP—induced-protein-10 also— IFN γ-induced pr; pr—protein; Pre.—predictive; couple—coupled; Neutro—neutrophil; Attrac/t—attracting; induct—induction; predict—predictive; COVID—SARS-CoV2-virus; TNFα—tumor necrosis factor alpha; IL-1—interleukin-1; IFN-γEo—Interferon gamma Eotaxin; chem—chemokine; M-trophic HIV—macrophage-tropic human immunodeficiency virus-1; Mel—melanoma; IFN—interferon; COX2—cyclooxygenase 2; slanDC—6-sulfo LacNAc^+^ dendritic cells; Jak—Janus Kinase; TYKinase—tyrosine kinase; T-reg—T-cell regulated; IL10-R’s—interleukin 10 receptors; DC—dendritic cells; T—T-cells; Th1—Theta-1 cells; Stim—stimulatory; prod—production; Het—hetero; wbc—white blood cells; Leuco—leucocytes; H&T cells—helper T0 cells; IgE—immunoglobulin E; Th—theta-1 cells; prog—prognostication; IL-1ra—regulatory adaptive (response); Tri—3 TNF-α molecules; T-membrane—trans-membrane.

**Table 4:** Comparison of statistical analysis.

Comparison	TTest/p 1-Tail t *	COVID *n*	PASC *n*	PASC R:p	COVID r:p
Eotaxin vs. Eo-3	None; Eo 0.11*0.05	27	12	−0.4; 0.16	0.006; 0.16
IFN-γ vs. IL-1β	Tr0.07; r-0.1p0.6	25	16	0.36; 0.17	0.3:p0.122
IL-8 vs. IP-10	<0.02 IP10; IL8 p0.9	24	16	0.13:0.7	0.22; p0.26
MCP1vs. MCP4	None; r = 0.6; *p* < 0.0001	25	16	0.2; *p* = 0.54	0.68; <0.0001
IL-8 vs. TNFα	<0.02;	25	16	0.2; *p* = 0.47	−0.1; 0.62
MDC vs. MIP-1α	0.3	25	16	−0.18; *p* = 0.6	0.5; <0.01
MIP-1β vs. TARC	0.7	25	16	0.6; *p* < 0.018	0.5; <0.015
IL-1β vs. IL-10	0.07 IL-1b;	11 il10 8	16/il7	0.13; *p* = 0.7	−0.3; *p* = 0.3
IL10 vs. IL12p70α	0.3	8	7	r = 0.76; *p* < 0.02	r = 0.5; *p* = 0.9
IL-2 vs. IL4	0.5	8	4	0.2; *p* = 0.8	−0.1; *p* = 0.8

TTest—Students *t*-Test; Tr—trend; r—regression coefficient; *p*—probability value. * Asterix is to make clear that we used a 1-tailed test for Eotaxin vs, Eo-3 and not a 2-tailed as for every other comparison.

**Table 5:** Demographics, signs, and symptoms of the various chronic viral syndromes.

Demo/Symptoms/Sign	Chronic Atypical Illness	Chronic Fatigue/ME	Long COVID
Age (years)	24.6 ± 5.0	Peak 20–25	45 ± 15 (France) 43.8 ± 13.4 (US)
Sex (% male)	28.6	25	23 44 France vs. US
Illness Duration (months)	19.14 ± 5.15 then ongoing	36–108	1–3 subacute/ongoing >3 chronic
Prevalence %	Unknown	Estimated 0.1–0.76%	32.6 persist19worse
Chronic Fatigue/malaise	71.4	100 by definition	39–63
Mood/cognitive impaired	28.6	46.89 (0.92) Illness severity scores	23–40
Myalgia/joint pain	14.3	73	2–19.6/9–27.3
Weakness	43	95	63
GI symptoms %	28.6	82–86 depending on criteria	0.9–10.5 diarrhea fecal shedding/dysbiosis
Thyroidopathy	28.6	16.3	Worsening control
Allergy history	43	50–75	#OR 1.67(CI 1.04 to 2.67)
Lymphadenopathy %	71.4	17	Reported 13.8–19
Fever-subjective	71.4	46–57 low grade fever DOC	0–0.9
Hepatomegaly	28.6	0	NA
Splenomegaly	71.4	0	NA
Lymphocytosis	71.4	20	NA
2–5oligoAsynthetase %	100	59	NA
IgM VCA EBV positive %	100	57 in a special subset	66.7 *p* < 0.001
IgG VCA EBV positive %	100	90 general population	Not associated
EBV early antigen R %	100	33.3	ND
EBV early antigen D %	100	17.6 & 79(a special subset)	66.7 *p* < 0.001
EBV Nuclear Antigen %	100	14.8	Not associated
CMV IgM %	28.6	0 in a special subset	Not associated
CMV IgG %	100	30 (>1:320)	Not associated
Heterophile Ab %	14.3	NA	ND
References	[2,3,4,6,8,9,10]	[11,12,13,14,15,16,17,18]	[19,20,21,22]

Long COVID has unique features that are not necessarily shared by the other syndromes, such as the following: Hair loss, 20–22%; Dyspnea, 42–66%; Taste and smell, 7–22.7%; Headache, 1.8–17.8%; Chest pain, 20%. Note that similar persistent symptoms were described for the SARS-CoV and MERS pandemics.NA-not applicable.

**Table 6:** Summary of demographic data.

Parameter	Chronic Fatigue	Controls	Statistical *p* Value (CI)
Number	13	53	N/A
Age (Years ± SD)	44.77 ± 15.94	55.17 ± 7.92	*p* < 0.04
% African American	53.9	69.2	*p* = 0.34
% Male	61.5	82.7	*p* = 0.13
Overweight %BMI > 28 kg/m^2^	25	62.8	OR21[0.90–490.14); *p* < 0.041
% Survival	92.3	96	*p* = 0.89
% Helicobacter pylori infection	16.7	34.4	*p* = 0.39
% Smokers	67	38.5	*p* = 0.11
% Alcohol Imbibers	54	36	*p* = 0.3

CI—Confidence interval; OR—odds ratio.

**Table 7:** Additional parameters of neoplasia, inflammation, kidney function, medication and history of tonsillectomy.

Parameter	Chronic Fatigue	Controls	Statistical *p* Value (CI)
Cumulative adenomas x ± sd	1.00 ± 1.48	2.89 ± 4.45	*p* < 0.002
Adenomas/colonoscopy x ± sd	0.08 ± 0.28	0.56 ± 1.22	*p* < 0.014
Inflammatory field colon x ± sd	0.417 ± 0.589	1.338 ± 0.555	*p* < 0.045
Blood Creatinine x ± sd mg/dL	0.939 ± 0.198	1.129 ± 0.256	*p* < 0.016
Proton Pump Inhibitors taken	50%	18.2%	*p* = 0.11
Calcium Channel Blockers/ACEi	25%/37.5%	42.8%/44.1%	*p* = 0.4/*p* = 1
History of Tonsillectomy	42.9%	0%	OR21[0.9–490.14]; *p* < 0.032

OR—odds ratio; ACEi—angiotensin converting enzyme inhibitor. The inflammatory field is a mean of graded chronic inflammatory changes in the lamina propria of colonic biopsies stained by hematoxylin noted by the pathologist assigned a value 0 (no inflammation) to 1 (increased lymphocytes), 2 (lymphoid aggregates), 3 (lymphoid follicles), numbers are similar with those in Table 6.

**Table 8:** Summary of Selected Long COVID Publications.

Paper (Cite #)	Patient Number	Publication Type	Control	Conclusions
Anaya et al. [27]	100; 10Vaccinated	Cross-sectional/MA	Acute vs. LC	>Arthralgia in LC
Becker et al. [28]	90; at 90 and 365 days	Prospective	LC vs. non-LC	>Severe initial; days
Haran et al. [29]	164; 13<; 14 > 4 wks	Prospective Dysbiosis	LC vs. non-LC	Prevatella/Veillonella *
Fogarty et al. [30]	50; median 68 days	Coagulation factors	17 normal	Increased; older LC **
García-Abellán [31]	146; 5.5% deaths	Prospective	None	Weak ab; Ꝗ, severity
Vanichkachorn [32]	100; mean 93 days	Descriptive > wks LC	None	80% fatigue; 59%neuro
Moreno-Pérez [33]	141; 34/66%mild/s	Prospective@77 days	None	No markers for LC
Ludvigsson [34]	5; 4 girls; 1p-myoC	Literature review	None	Sx for 4–8 months
Wong [35]	21 studies LC and CFS	Literature review	Compare Sx	86%Sx shared ME/CFS

Legend. #—Paper citation number; MA—Meta-analysis; LC—Long COVID; GI—diarrhea; Initial—from time of acute COVD19; Days—extended hospital stays; * also seen in CFS; ** Von Willebrand factor blood antigen, pro-peptide, factor VIII, thrombomodulin. ab—antibody, Ꝗ—female; Sx—symptoms; ME/CFS—Myalgic encephalitis/chronic fatigue syndrome; 1p-myoC—peri-myocarditis admission.

## Data Availability

The data presented in this study are available on request from the corresponding author due to Data Transfer Agreement. This term means a written agreement between the provider and the recipient of data that are transferred from one to the other. It defines what data may be used, how the data will be used, who may access and use the data, how the data must be stored and secured, and how the recipient will dispose of the data after completion of the research.
